# Real-time sign language detection: Empowering the disabled community

**DOI:** 10.1016/j.mex.2024.102901

**Published:** 2024-08-08

**Authors:** Sumit Kumar, Ruchi Rani, Ulka Chaudhari

**Affiliations:** a Symbiosis Institute of Technology, Pune Campus, Symbiosis International (Deemed University), Pune, Maharashtra 412115, India; b Department of Computer Engineering and Technology, School of Computer Engineering and Technology, Dr. Vishwanath Karad MIT World Peace University Pune, Pune, Maharashtra 411038, India

**Keywords:** Sign Language (SL), Disabled, Transfer learning, Convolutional neural networks (CNNs), VGG16 model, Pre-trained models, Classification, VGG16 with an attention mechanism

## Abstract

Interaction and communication for normal human beings are easier than for a person with disabilities like speaking and hearing who may face communication problems with other people. Sign Language helps reduce this communication gap between a normal and disabled person. The prior solutions proposed using several deep learning techniques, such as Convolutional Neural Networks, Support Vector Machines, and K-Nearest Neighbors, have either demonstrated low accuracy or have not been implemented as real-time working systems. This system addresses both issues effectively. This work extends the difficulties faced while classifying the characters in Indian Sign Language(ISL). It can identify a total of 23 hand poses of the ISL. The system uses a pre-trained VGG16 Convolution Neural Network(CNN) with an attention mechanism. The model's training uses the Adam optimizer and cross-entropy loss function. The results demonstrate the effectiveness of transfer learning for ISL classification, achieving an accuracy of 97.5 % with VGG16 and 99.8 % with VGG16 plus attention mechanism.

• Enabling quick and accurate sign language recognition with the help of trained model VGG16 with an attention mechanism.
• The system does not require any external gloves or sensors, which helps to eliminate the need for physical sensors while simplifying the process with reduced costs.
• Real-time processing makes the system more helpful for people with speaking and hearing disabilities, making it easier for them to communicate with other humans.

## Graphical abstract

**Figure:**
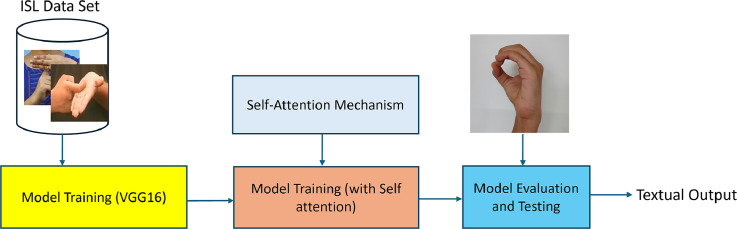

Specifications table

**Table:** 

Subject area:	Deep Learning, Pre-trained model, Real-time image processing
More specific subject area:	VGG16, attention mechanism
Name of your method:	VGG16 with an attention mechanism
Name and reference of original method:	NA
Resource availability:	Jupyter Notebook, Python IDLE, Pytorch

## Background

The ability to communicate effectively is fundamental to human interaction. However, for individuals with disabilities such as hearing or speaking impairments, this can be a significant challenge. Sign language acts as a link by enabling these individuals to communicate with others. However, a significant communication gap exists between sign language users and those unfamiliar. Thus, daily interactions, access to public services, and overall quality of life are hindered due to this gap.

This research aims to fill the communication gap more effectively and affordably. There is a need for improvement in the present work [1] in the literature in two major areas: accuracy and real-time application. Many solutions have used different machine learning and deep learning techniques, such as Convolutional Neural Networks (CNN) [[2], [3], [4], [5], [6]], Support Vector Machines (SVM), K-nearest neighbors (KNN), etc. However, these techniques do not achieve high accuracy and are unsuitable for real-time use. Even some systems depend on additional hardware, like gloves with sensors, which makes the system costly and difficult to use. Thus, the proposed work provides a more accurate, affordable, and real-time solution for Indian Sign Language (ISL) recogni-tion [7].

This work uses a pre-trained VGG16 [9,10] Convolutional Neural Network (CNN) [[11], [12]] with an attention mechanism. The VGG16 model performs well in image classification tasks as it can extract the more complicated details of hand poses in sign language. The attention mechanism enhances the model performance by focusing on the relevant areas of the input image. Moreover, the attention mechanism highlights the essential features, such as the specific hand shapes and movements in sign language, by reducing the amount of background information that is not important. In this way, the ability of the model of classification is enhanced. So, the attention mechanism helps reduce the model's sensitivity to noise and variations in the input images, which results in increased classification accuracy. By using transfer learning, the pre-trained VGG16 model is fine-tuned to recognize 23 distinct hand poses in ISL, achieving high accuracy while utilizing low computational resources.

Also, the real-time testing of the proposed system confirms its practical useability and will benefit those suffering from speaking and hearing disabilities. It immediately translates sign language into text and helps in smoother and more efficient communication. Hence, it can enhance the day-to-day interactions of sign-language users because they can now communicate more easily with non-sign-language users.

By utilizing advanced deep learning techniques and focusing on real-time processing, this research aims to create an accessible, affordable, and highly accurate sign language recognition system. This system can improve the quality of life of disabled persons by enabling more effective and uninterrupted interactions with the outside world. Table 1 compares various sign language recognition studies comprehensively, highlighting the diversity in sign languages, datasets, methodologies, and accuracies achieved. Also Cost-effective SLR systems are critical for commercial use, with various methods reviewed for practical implementation [13]. Moreover, Few-shot learning shows promising results in cross-lingual SLR, enabling the recognition of new signs with limited data [14]. Research on Indian Sign Language (ISL) has improved accessibility by tackling gesture recognition and skin segmentation [15]. Federated learning has enhanced Bengali Sign Language recognition while maintaining user privacy [16].

**Table 1:** Comparison of various sign language recognition studies.

Refs.	Sign Language	Dataset Used	Methodology	Main Techniques	Accuracy	Focus
[2]	American Sign Language (ASL)	ASL datasets	Desktop application for real-time sign language recognition	CNN	96.30 %	Real-time text conversion
[3]	Various	Custom dataset	Automated Sign Language Detection and Classification	MobileNet, CNN, LSTM, MRFO	99.28 %	Hybrid deep learning model, optimal hyperparameter selection
[4]	Pakistan Sign Language (PSL)	Custom dataset	Hand gesture recognition	CNN, SIFT	98.74 %	Uses Kinect sensor, impressive accuracy, feature extraction with SIFT
[5]	Chinese Sign Language	Custom dataset	Wearable sign language recognition system	CNN, stretchable strain sensors, IMUs	95.85 % (words), 84 % (sentences)	Combines multiple sliding windows for sentence recognition
[6]	American Sign Language (ASL) & British Sign Language (BSL)	Custom dataset	Recognition using computer vision algorithms and neural networks	CNN, Mediapipe keypoint detection	CNN= 97.24 % RNN using LSTM= 87.4 % DTW-KNN =83.7 %	Real-time feedback integration, flexible for various settings
[7]	Assamese Sign Language	Custom dataset	Recognition system for Assamese Sign Language	MediaPipe, feed-forward neural network	99 %	Focus on the local dialect, uses MediaPipe for landmark detection
[8]	Various	CSL-500, LSA64 datasets	Signer-independent learning method	GRU, MLP	98.75 %	Addressing overfitting, using mask replacement method for data augmentation
[11]	Indian Sign Language (ISL)	Custom dataset	Recognition of ISL gestures	3D CNN	88.24 %	Focus on both static and dynamic gestures, diverse dataset conditions

## Method details

**Fig. 1:**
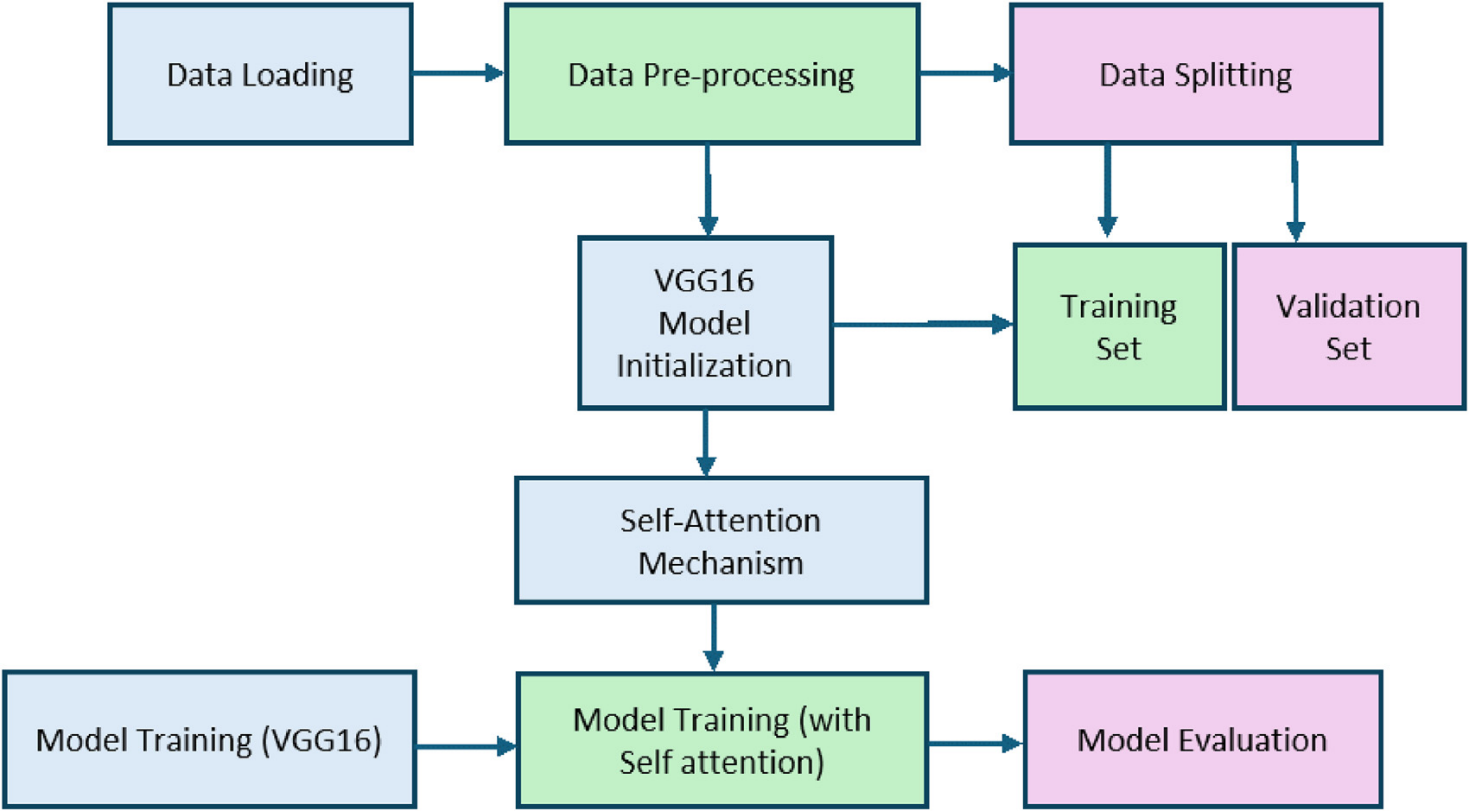
Methodology of the proposed work.

The proposed work provides a systematic approach for training and evaluating a sign language classification model upgraded with a self-attention mechanism, as shown in Fig. 1. Initially, the sign language dataset is loaded and preprocessed. The dataset has images of signs corresponding to different letters. Preprocessing steps are required to prepare it for model input. It includes resizing the images and converting them into tensors, etc. The dataset is split into training and validation sets afterward. The VGG16 model is trained on the dataset, and further self-attention mechanism is used to enhance accuracy. The Adam optimizer and cross-entropy loss function are used over multiple epochs to optimize the model parameters during training. Now, the model is evaluated on a validation set of the datasets, and the performance of the model is calculated in the form of accuracy, precision, recall, and F1-score. A confusion matrix is created so that every sign class can comprehensively viewed in the model's classification. Furthermore, the model was tested on real-time gestures, and it successfully recognized the gestures correctly, demonstrating its practical applicability.

## Dataset

**Fig. 2:**
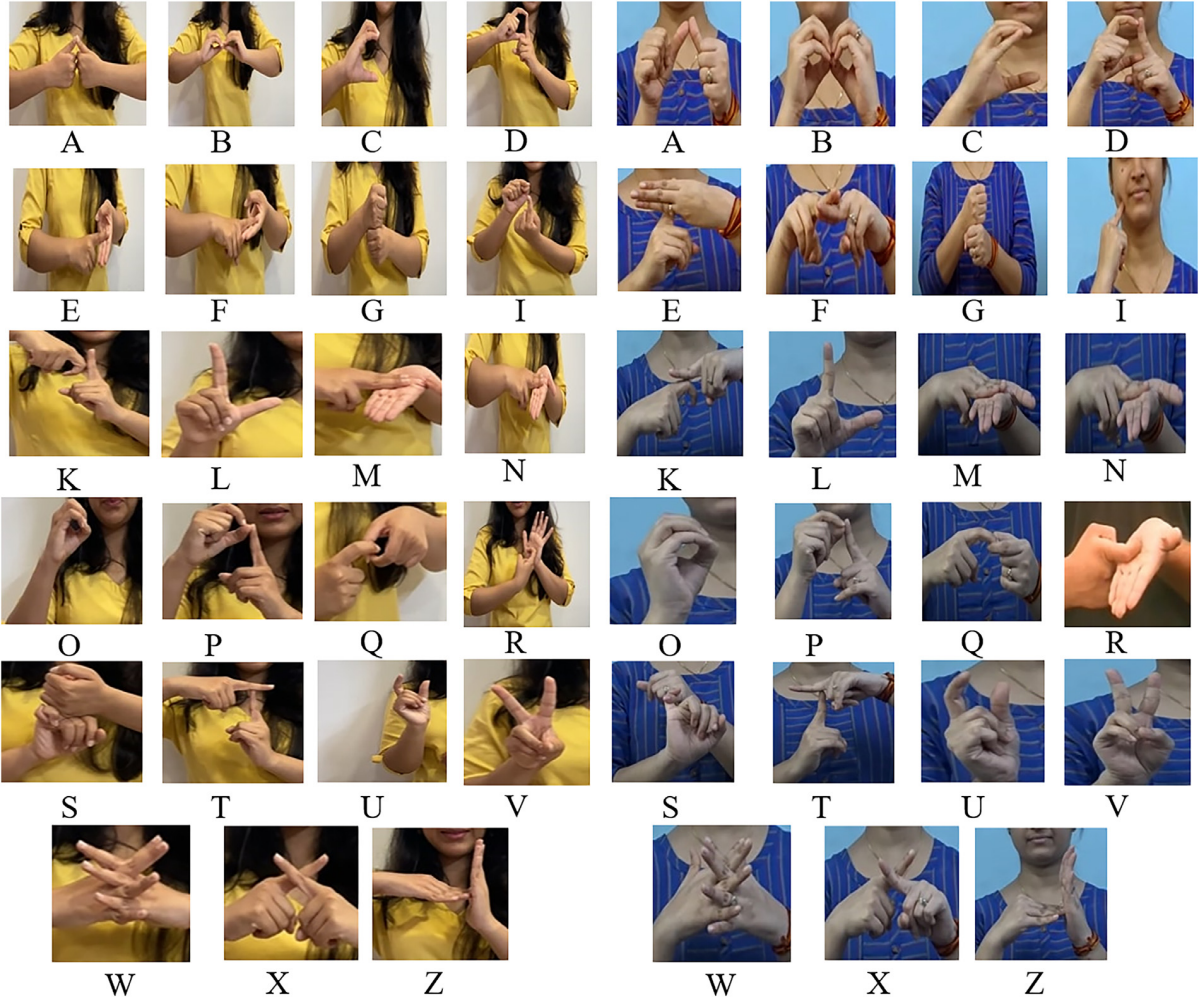
Sample Image dataset used for the system training and validation.

Data Acquisition is the operation of capturing the images of the hand images representing different signs. The system uses a publicly available data set for training and testing. This dataset contains a total of 23 classes. The signs used in this dataset are A, B, C, D, E, F, G, I, K, L, M, N, O, P, Q, R, S, T, U, V, W, X, Z. The dataset includes various collections of ISL gesture images that were captured and processed to simulate real-world scenes. The data set is taken from Kaggle (https://www.kaggle.com/datasets/soumyakushwaha/indian-sign-language-dataset). The original images feature many gestures representing many sign language expressions. Each image adds a noise-like, blurry, messy, colorful background to provide real-world communication scenes. Fig. 2 shows sample gesture images of all 23 signs from the data set. The dataset contained images of 23 different sign language gestures from A to Z, excluding the letters H, J, and Y. The total images in this are 702 images with 126 × 126 pixels.

• Image Processing
  Image preprocessing is fundamental before using images for tasks like sign language recognition. Like cleaning and segregating data before analysis, preprocessing ensures that the images are in a format needed for the computer vision model. This may involve removing noisy backgrounds or resizing images to all the same sizes. Preprocessing is an important step because it increases the quality of the information the model receives. By reducing noise and focusing on relevant image features, the model can learn more effectively and achieve better recognition accuracy.
• Normalization
  Images are usually stored in the format of grids and pixels, where each pixel is represented by its numerical value and its color intensity. These values are dependent on the image format. Challenges faced by CNN because of using raw data:
  ∘ Inconsistent data
  ∘ Activation Function
  The above various challenges can be solved by performing normalization. This process works by rescaling the pixel values in the image data to a similar range. Here, the function ToTensor() mainly converts the numerical pixel values (mostly between 0 and 255 for grayscale or a wider range for RGB images) into a PyTorch tensor with 0 as minimum and 1 as maximum.
• Resizing
  Using data without resizing the information appropriately may cause various issues when preparing the CNN, such as computational complexity and difficulties in learning patterns.
  Therefore, we use the Resizing of the images to ensure all the images are in a uniform size. The size used in this system is (224,224). We must correctly choose the image dimensions for the specific architecture being used. The VGG16 used in our system are pre-trained on a specific size, i.e. 224 × 224. Using the same size as the pre-trained model ensures compatibility with the pre-trained model.

## Model architecture

**Fig. 3:**
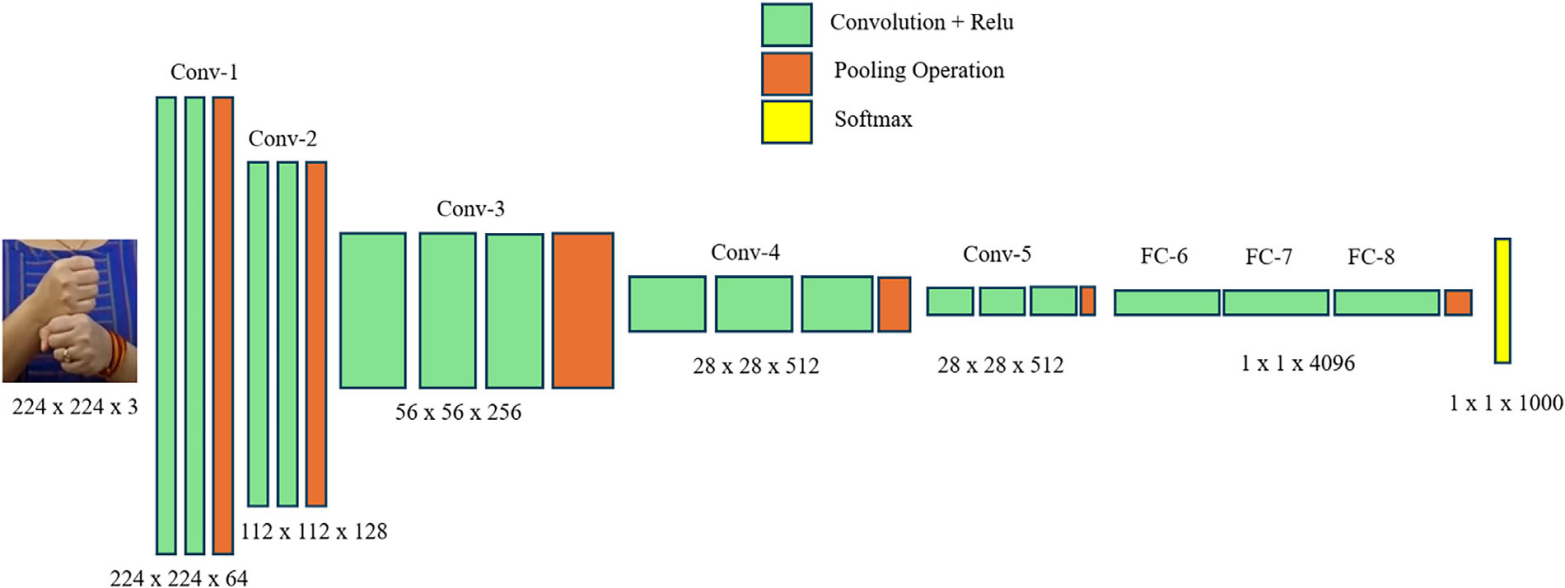
VGG16 Convolution layers architecture.

• VGG16
  The primary objective of this study is to enhance the performance of sign language gesture classification using deep learning techniques. The pre-trained VGG16 model, which has already learned rich feature representations from the ImageNet dataset, is a strong starting point for transfer learning. In the sign language recognition system, VGG16 is the main backbone of the system for feature extraction and classification. VGG 16 is a pre-trained CNN model. VGG16 uses convolution layers, which in turn help with the extraction of features. They essentially slide small filters across the image, and each image detects patterns or features. During the training of the VGG16, a large dataset is used, and they start by identifying low-level features like edges and lines and then combine them into more complex features. VGG16 uses pooling layers between the convolution layers. These layers reduce the image size while still preserving its important features. The output is a compressed feature vector capturing important features learned from the image. The VGG16 model is a deep convolutional neural network comprising 16 layers, as shown in Fig. 3. It is pre-trained on the ImageNet dataset and widely used for various image classification tasks. The architecture of the VGG16 model used in this study is as follows:
  ∘ Convolutional Layers: The model includes a series of convolutional layers with increasing depth (number of filters) and ReLU activation functions.
  ∘ Pooling Layers: Max-pooling layers are used to reduce the spatial dimensions of the feature maps.
  ∘ Fully Connected Layers: The final part of the model consists of three fully connected layers, with dropout layers to prevent overfitting.
  ∘ Output Layer: The output layer is modified to match the number of classes in the sign language dataset.

Activation functions like Relu are given in Eq. (1), and a nonlinear activation function is used to help learn more complex data, compute and learn, and provide accurate predictions. This is one of the most popular activation functions used in hidden layers of neural networks. Relu is less computationally expensive than tanh and sigmoid activation functions.

(1) f(x)=ReLU=max(0,z)

The other type of activation function used in the convolution layers is the SoftMax activation function shown in Eq. (2). This layer is useful for classifying the images in the last layers of the convolution. The other advantage of this function is that this function can handle multiple classes.

(2) $$f\left(x\right)=\text{softmax}\left(z_{i}\right)=\frac{e^{z_{i}}}{\sum_{j=1}^{N}e^{z_{j}}}$$

where **z** is the vector of raw outputs from the neural network, the value of e ≈ 2.718, The i^th^ entry in the SoftMax output vector SoftMax(**z**) can be thought of as the predicted probability of the test input belonging to class i.

**Fig. 4:**
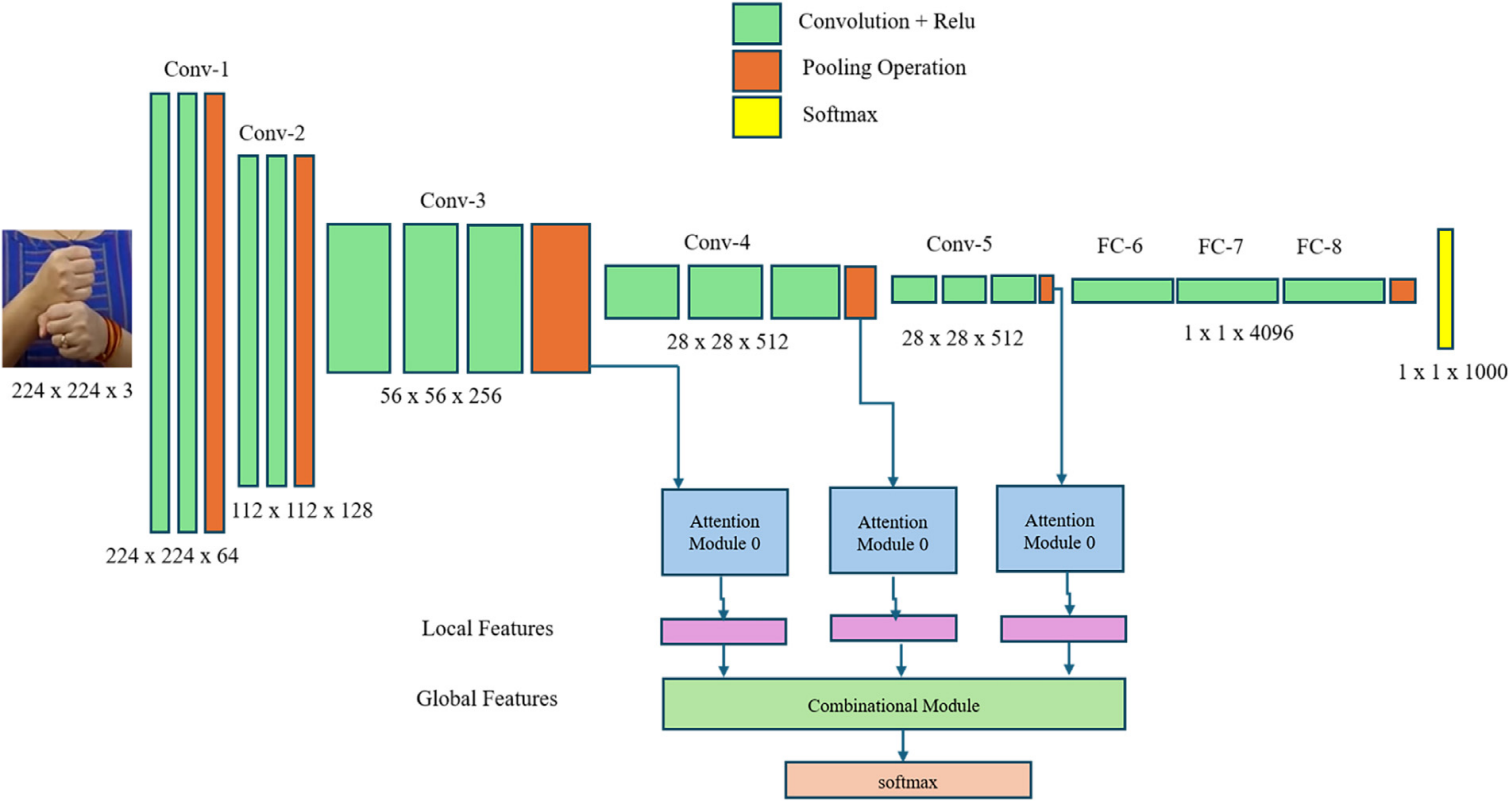
VGG16 with attention mechanism architecture.
∘ Fully Connected Layers: Like the VGG16 model, the final layers are fully connected layers with dropout for regularization.
∘ Output Layer: The output layer is adjusted to match the number of classes in the dataset.
∘ Incorporating attention mechanisms into the VGG16 model aims to improve classification accuracy further. Attention mechanisms enable the model to focus on the most relevant parts of an image, which is particularly beneficial for tasks like sign language classification, where subtle differences in hand gestures are crucial. By integrating attention mechanisms, we hypothesize that the model will better capture these important details, leading to improved performance.

• VGG16 with Attention Mechanisms
  Attention mechanisms are integrated into the architecture to enhance the performance of the VGG16 model. Attention mechanisms allow the model to focus on the most relevant parts of the input image, potentially improving classification accuracy. The architecture of the VGG16 with Attention model is shown in Fig. 4.
  ∘ Base VGG16 Model: The model's core remains the same as the standard VGG16, utilizing the pre-trained convolutional layers.
  ∘ Attention Blocks: The attention block is responsible for refining the feature maps by focusing on the most informative parts of the image. It operates as follows:
    ∘ Local Features: The initial convolutional layers extract the local features.
    ∘ Global Features: These are obtained by applying a global average pooling operation.
    ∘ SoftMax: The attention map is generated by applying a SoftMax operation to the global features. This map highlights the most relevant parts of the input.
    Attention blocks are inserted after certain convolutional layers. Each attention block consists of:
    ∘ Conv1: A convolutional layer that reduces the number of channels.
    ∘ Attention Calculation: Softmax is applied to the output to generate an attention map.
    ∘ Conv2: Another convolutional layer to scale the attention map back to the original number of channels.
    ∘ Element-wise Addition: The attention map is added back to the input feature map to produce the final output of the attention block.

## Method validation

The validation starts with the performance of several pre-trained convolutional neural network (CNN) models to determine the most suitable one for real-time sign language detection in Indian Sign Language (ISL). The models compared are VGG16, ResNet50, InceptionV3, and MobileNetV2. The performance metrics used for comparison are accuracy, precision, recall, and F1-score. In the dataset, 600 random images from 702 images are taken for training, and 102 are used as a validation set, so 14.5 % of the total dataset was used for validation. The comparison of pre-trained models on the sign language dataset is shown in Table 2.

**Table 2:** Comparison of several pre-trained convolutional neural network (CNN) models.

Model	Accuracy	Precision	Recall	F1-Score
ResNet50	92.3 %	91.5 %	93.9 %	92.7 %
InceptionV3	90.8 %	92.8 %	91.1 %	92.9 %
MobileNetV2	89.0 %	90.1 %	91.4 %	91.2 %
VGG16	97.5 %	96.8 %	97.2 %	97.0 %

Based on this comparison, VGG16 is the most effective model for our real-time ISL detection system, providing a high accuracy, precision, recall, and F1 score alongside good feature extraction capabilities and the benefits of transfer learning. After selecting VGG16, it is trained for 30 epochs with and without an attention mechanism. Key performance metrics such as accuracy and loss are recorded for training and validation datasets during training. The optimization of hyperparameters is conducted to achieve the best possible model performance.

• VGG16 Model: The base VGG16 model serves as the benchmark. It is trained using a learning rate of 0.001 and a batch size 32.
• VGG16 with Attention Mechanism: The attention mechanism is integrated into the VGG16 architecture to enhance the model's focus on relevant features within the input images. This modified model also exhibites substantial improvements, achieving similar peaks in training and validation accuracies and demonstrating a more nuanced understanding of the sign language gestures.

### Performance metrics

**Fig. 5:**
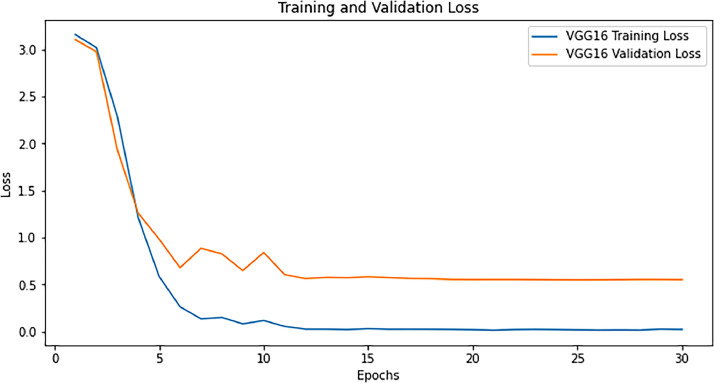
Training loss and validation loss for VGG16.

**Fig. 6:**
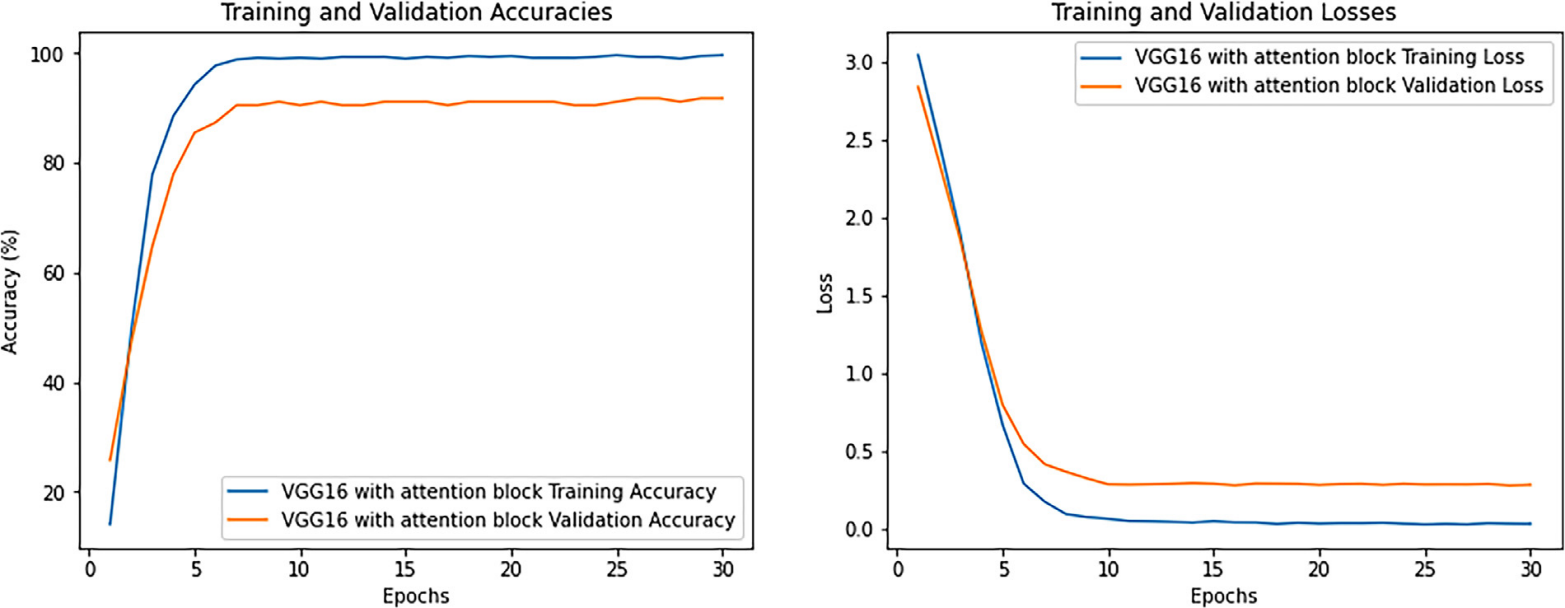
Training loss and validation loss.

After testing our model on the validation set, the following results are acquired with the graph plotted for training loss and validation loss for VGG 16 in Fig. 5 and training loss, validation loss, training accuracy, and validation accuracy for VGG16 with attention mechanism in Fig. 6.

Precision, recall, and F1-score are computed for each class to assess the model performance, as shown in Table 3. The classification report for the sign language recognition system shows a high overall performance with an average precision of 0.978, a recall of 0.984, and an F1-score of 0.973. The individual sign classes demonstrate consistent accuracy, with many achieving perfect scores 1.0 across all metrics. Notable exceptions include signs B, D, F, K, O, P, T, X, and Z, with slightly lower scores in certain metrics. Despite these variations, the model maintains robust performance, effectively recognizing most signs with high precision, recall, and F1 scores. This highlights the model's capability to accurately identify sign language gestures, contributing significantly to the advancement of sign language recognition technology.

**Table 3:** Precision, recall, and f1 score of the proposed model.

Sign	Precision	Recall	f1-score
A	1	1	1
B	1	1	0.94
C	1	1	1
D	1	1	0.94
E	1	1	1
F	1	0.86	0.94
G	1	1	1
I	1	1	1
K	0.9	1	1
L	1	1	1
M	1	1	1
N	1	1	1
O	0.8	0.9	1
P	1	0.88	0.95
Q	1	1	1
R	1	1	1
S	1	1	1
T	0.8	1	1
U	1	1	1
V	1	1	1
W	1	1	1
X	1	1	0.73
Z	1	1	0.89
**Average**	**0.978261**	**0.984348**	**0.973478**

Based on this comprehensive evaluation, VGG16 stands out as the most effective model for our real-time ISL detection system, providing a balance of high accuracy, precision, recall, and F1 score alongside robust feature extraction capabilities and the benefits of transfer learning. The validation results indicate that the attention mechanism significantly enhances the VGG16 model's performance, particularly in distinguishing between visually similar gestures. After incorporating the attention mechanism, the performance of our model improved across all metrics. The comparative results are shown in Table 4 below:

**Table 4:** Comparison of the Base Model VGG16 with an attention mechanism.

Model	Accuracy	Precision	Recall	F1-Score
VGG16	97.5 %	96.8 %	97.2 %	97.0 %
VGG16 + Attention Mechanism	99.80 %	97.80 %	98.40 %	97.30 %

**Fig. 7:**
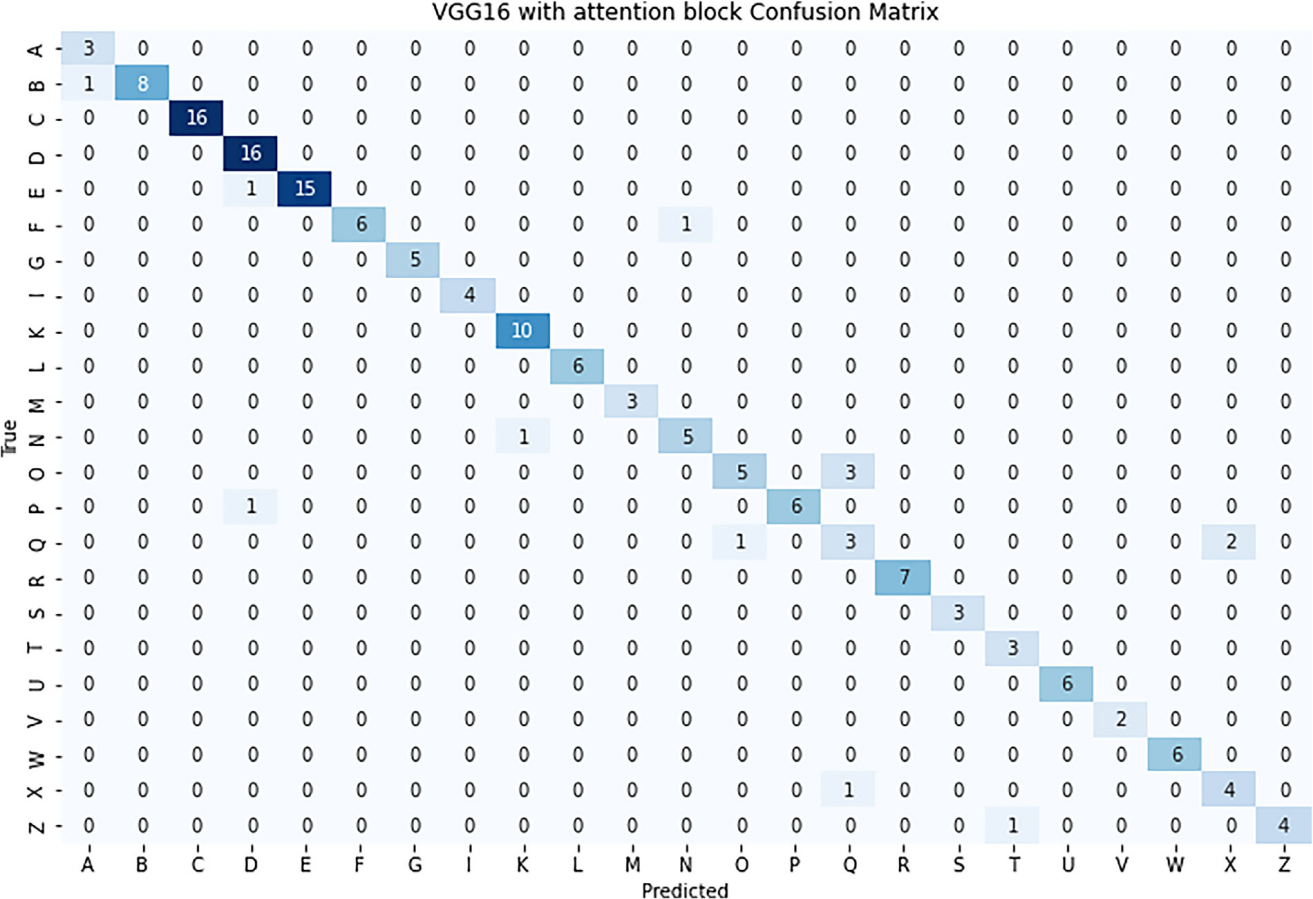
Confusion Matrix of the model.

The confusion matrix for the VGG16 model with the self-attention mechanism, as shown in Fig. 7, provides a comprehensive view of the model's classification performance across various sign language gestures.

## Real-world testing

**Fig. 8:**
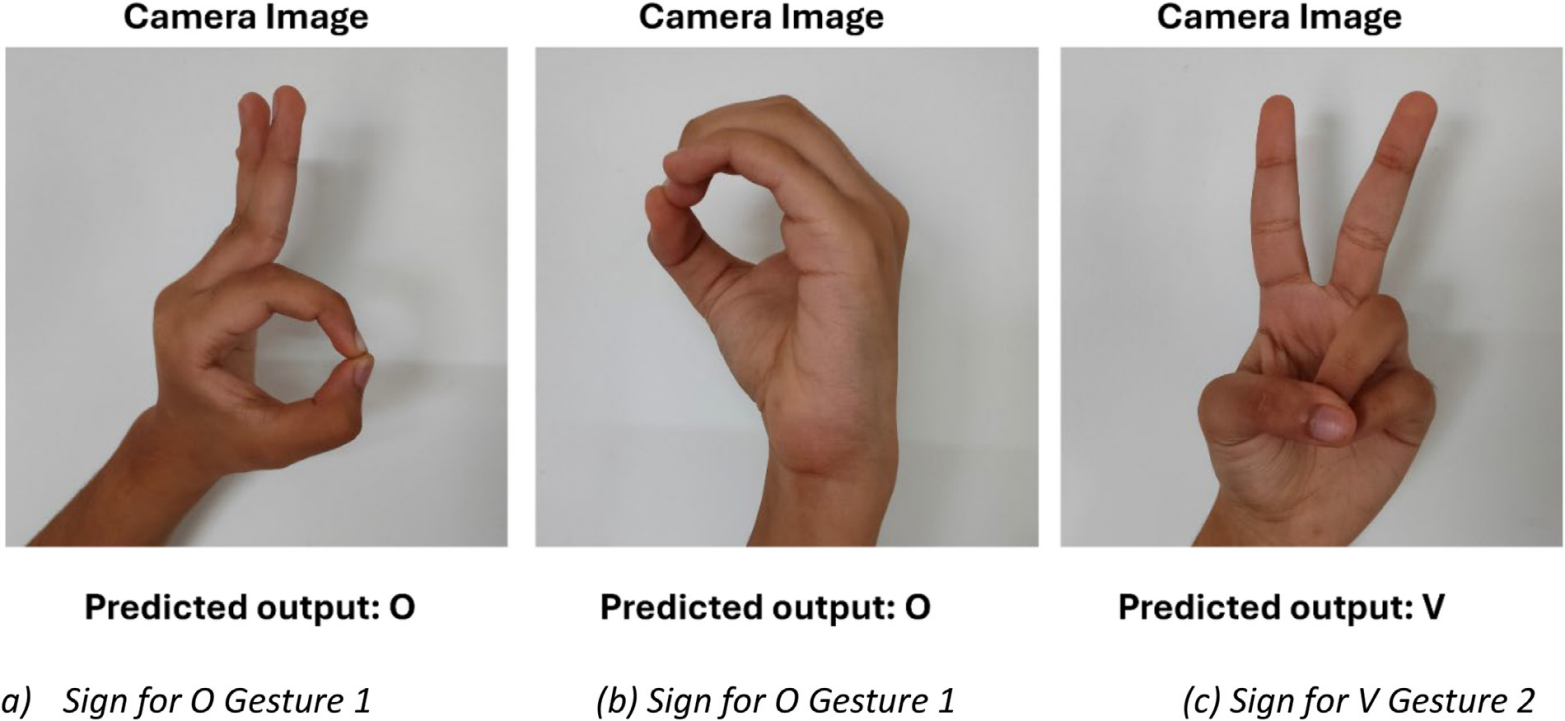
Real-time predicted outputs of the model.

Apart from the dataset images, the proposed model is tested with real-world sign language gestures to evaluate its practical applicability. The model can accurately identify and classify the signs, which shows that this model is suitable for real-time scenarios. The images in Fig. 8 are the Real-time images captured for prediction with the correct outputs predicted at the bottom of the image. The outputs are the signs for V and O with two different gestures for O.

Table 5 provides a comparative analysis of the proposed model with existing works in sign language recognition. The proposed model, which incorporates a VGG16 architecture enhanced with an attention mechanism, achieves an impressive accuracy of 99.8 %, surpassing most existing methodologies. The proposed model's integration of an attention mechanism with VGG16 achieves the highest accuracy. It demonstrates robustness in real-world sign language gestures, significantly advancing the practical applicability of sign language recognition technology.

**Table 5:** Comparison of the proposed model with existing work.

Refs.	Methodology	Main Techniques	Accuracy	Important features
[11]	Recognition of ISL gestures	3D CNN	88.24 %	Focus on both static and dynamic gestures, diverse dataset conditions
[17]	Real-time hand pose and gesture recognition	Grid-based feature extraction, k-NN, HMM	99.7 % (static), 97.23 % (gestures)	It uses a smartphone camera, no external hardware, and high accuracy in static poses and gestures.
[18]	SLR using key frame extraction and two-stream networks	Key frame extraction, two-stream networks	State-of-the-art (CSL–500), competitive (LSA64)	Efficient summarization of video information, Full-hand two-stream network with attention blocks
[19]	ISL gesture recognition	CNN	99 %	Addresses communication gap, high variability in hand shapes, custom dataset, high accuracy
[20]	Real-time sign language recognition system	YOLOv4, SVM with Mediapipe	YOLOv4: 98.8%, SVM: 98.62%	No pre-processing required, handles continuous gestures, proposed expert system for enhanced real-time accuracy
[21]	Real-time ISL recognition system	YOLOv3, Darknet-53	95.7% (static), 93.1 % (dynamic)	Recognizes both static and dynamic signs, tested in real-time, reduced computational time
[22]	ISL gesture translation	ResNet-50, InceptionNet V3	ResNet-50: 98.25 %, InceptionNet V3: 66.75 %	Custom dataset with diverse conditions, comparative study of deep learning architectures, ResNet-50 performs best
Our work	Real-Time Sign Language Detection with an attention mechanism	VGG16 with an attention mechanism	99.8 %	Uses an attention mechanism with VGG and is also tested with real-world sign language gestures to assess their practical applicability

## Limitations

Not applicable.

## Ethics statements

This research did not involve research on humans or animals, and no data is involved from social media platforms.

## CRediT authorship contribution statement

**Sumit Kumar:** Supervision, Funding acquisition, Validation, Visualization, Validation, Methodology, Writing – original draft. **Ruchi Rani:** Supervision, Writing – review & editing, Writing – original draft. **Ulka Chaudhari:** Conceptualization.

## Declaration of competing interest

The authors declare that they have no known competing financial interests or personal relationships that could have appeared to influence the work reported in this paper.

## Data Availability

No data was used for the research described in the article.
